# Comparing production metrics and financial efficiency in production-limited dairy herds

**DOI:** 10.3168/jdsc.2025-0848

**Published:** 2025-11-13

**Authors:** C.R. Church, L. Hayes, M.W. Overton, T.F. Duffield, D.F. Kelton

**Affiliations:** 1 Gordon S. Lang School of Business and Economics, University of Guelph, Guelph, ON, Canada N1G 2W1; 2 Zoetis Animal Health, Parsippany, NJ 07054; 3 Ontario Veterinary College, University of Guelph, Guelph, ON, Canada N1G 2W1

## Abstract

This retrospective observational study examined the relationships between production metrics and financial efficiency on dairy farms operating within a production-limited system in Canada. In such a system, production quotas serve as the primary constraint on herd expansion. Canadian financial advisors, including accountants and lenders, predominantly use earnings before interest, taxes, depreciation, and amortization (EBITDA) as their key success metric at the herd level. For comparative purposes, they often use quota holdings measured in kilograms of butterfat rather than cow numbers as the denominator. Data were collected from 42 Canadian farms for the years 2017 through 2021. Financial statements were standardized and adjusted to account for unpaid labor and dividends. Multivariable linear regression was used to describe the relationship between 17 commonly used metrics—including those related to milk production, reproduction, transition, replacements, and turnover—and the annual EBITDA per kilogram of butterfat quota (EBITDA/kg). Milk production per cow (ECM; 39 ± 4 kg/d) was not significantly associated with EBITDA/kg (Can$1,851 ± Can$1,000; Can$1 = US$0.72). Four metrics exhibited significant associations with EBITDA/kg. Specifically, labor as a percentage of revenue, purchased feed/kg of butterfat quota, and DIM demonstrated negative associations, whereas the percentage of the herd dry between 45 and 75 d exhibited a positive association. Earnings before interest, taxes, depreciation, and amortization serves as an indicator of the efficiency with which raw materials are converted into profit. The findings suggest that higher production levels are unrelated to increased milk production efficiency within a production-limited system at the herd level. Certain management factors, such as purchased feed/kg and labor, may result in suboptimal resource use. Conversely, factors such as DIM and days dry may influence the cow's efficiency in converting feed into milk at both the cow and herd level. Although the lack of association between production and EBITDA may not apply to non-production-limited markets, expanding the focus beyond production metrics to include financial efficiency enables advisors in all markets to identify management practices that may impede optimal milk production. This research highlights the importance of collaboration between production and financial advisors. Comparing key efficiency indicators alongside EBITDA/kg will ensure that producers concentrate on areas with the greatest potential for financial improvement.

## Highlights

• Canadian dairy farms operate under a supply-management system based on butterfat.
• Veterinarians and nutritionists use production per cow as their outcome target.
• Canadian financial advisors use profit per kilogram of butterfat quota as their primary outcome.
• Energy-corrected milk per cow was not associated with profit per kilogram of butterfat quota across herds.
• Improving biological and operational efficiency increases profit per kilogram.

## Graphical Abstract

**Figure:**
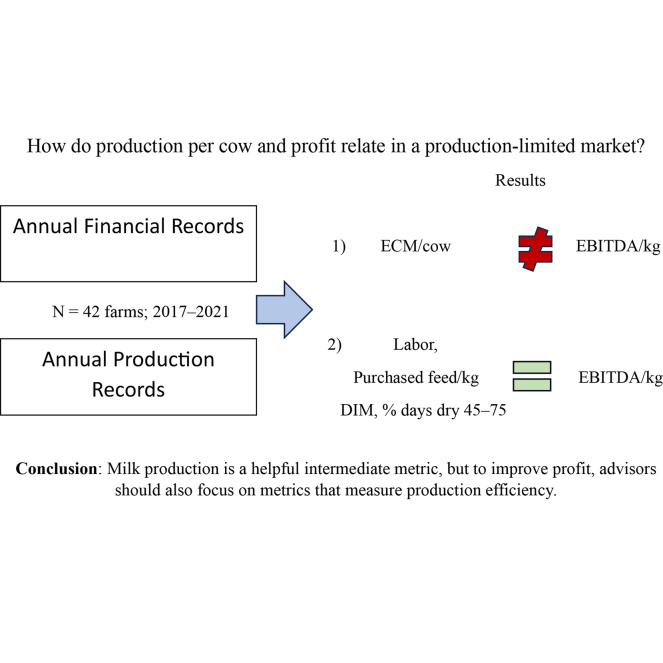
**Summary:** This retrospective observational study used annual farm financial and production records from 2017 to 2021 to examine the relationship between financial efficiency (earnings before interest, taxes, depreciation, and amortization [EBITDA] per kilogram) and production per cow across 42 Canadian herds. These herds operate under a supply-managed system where quotas are linked to butterfat. Veterinarians and nutritionists use production per cow to measure progress, similar to the global dairy community. However, Canadian lenders and accountants favor efficiency metrics per kilogram of butterfat quota as their primary indicators. The study found that EBITDA per kilogram was not associated with milk production per cow (energy-corrected milk [ECM]) within this dataset. However, EBITDA/kg was associated with other efficiency indicators, including labor, purchased feed per kilogram of butterfat quota, herd average days in milk (DIM), and the proportion of the herd dry between 45 and 75 days. Emphasizing both biological and financial efficiency enables production and financial advisors to collaboratively pursue shared outcomes for their dairy clients.

Producers depend on veterinarians and nutritionists for health and production advice, as well as on accountants and lenders for financial guidance. Each tries to act in the farm's best interest but may not prioritize profit as the main goal of the dairy.

Canadian dairy farms operate within a supply-managed or production-limiting (**PL**) system in which their milk sales are limited by the availability and cost of a market license (quota; CDC, 2022). Producers can fill their production allotment with either more lower-producing cows or fewer higher-producing cows. In both cases, revenue from calves, market cows, and other sources may vary; however, herd-level revenue from milk remains constant. Therefore, the goal is to increase profit by reducing expenses.

In non-PL markets, milk production per cow is the key factor affecting dairy revenue, and boosting production has been a central focus of the dairy community for decades (Balaine et al., 1981; Wilson, 2011). Dairy producers regard veterinarians and livestock nutritionists as their primary advisors (Mills et al., 2021). Recommendations from both can have substantial financial implications; however, producers often view discussions concerning comprehensive farm finances as outside their expertise and may limit their access to financial data (Hilkens et al., 2018). Consequently, veterinarians and nutritionists rely on production and health indicators to evaluate success. Nonetheless, monitoring production without accounting for expenses in any market can lead to financial illiteracy (Malcolm, 2004). Economic theory posits that increased production should be promoted, provided that the marginal revenue gains surpass the marginal costs (Macdonald et al., 2017). This requires someone to monitor these costs and revenues. Financial advisors, including accountants and lenders, also hold vital roles for producers. These advisors emphasize annual profit and financial efficiency as primary metrics but are infrequently consulted regarding production and feed costs.

Change efforts are more likely to succeed when production and financial advisors collaborate (Holden, 2017). However, this is problematic without a shared set of metrics and outcomes. Establishing common goals is crucial for aligning advisor recommendations and improving farm outcomes. The primary objective of this study was to investigate the relationship between standard production metrics and financial outcomes, and to foster shared objectives between production and financial advisors. Although other research has explored production and profit (Wilson, 2011; Hanrahan et al., 2018; Jayasundara et al., 2019), to our knowledge, none have compared production measures to operating efficiency on a per-kilogram basis in Canada (confinement housing, continuous calving, and PL).

A retrospective observational study was conducted on 42 Canadian farms from 2017 to 2021. Farms were invited to participate via provincial milk boards (Ontario, Manitoba, Alberta) and advisory groups (Ontario Association of Bovine Practitioners, Atlantic Association of Bovine Veterinarians). For inclusion, farms had to be located in Canada, fluent in English, enrolled in milk recording (Lactanet, Guelph, ON, Canada), have accrual-based annual financial statements, and receive at least 90% revenue from dairy sales (milk, salvage, calves, and replacements), as used in other dairy finance research (Jayasundara et al., 2019).

A consultation with 20 Canadian dairy financial advisors, comprising 12 lenders and 8 accountants, identified earnings before interest, taxes, and depreciation per kilogram of butterfat quota (**EBITDA/kg**) as their principal metric for evaluating the financial performance of dairy farms. Annual income statements were supplied by the farm's accountants and standardized for research according to Martin et al. (2017). Nonfarm income (including subsidies and one-time investments) was recorded after EBITDA. For remuneration, participants supplied the number of paid and unpaid full-time equivalent employees, and each farm was assigned a total wage expense based on a standardized hourly rate of Can$22 per hour. This was compared with the total disbursed via salaries and dividends. If actual wages fell below the theoretical amount, the deficit was incorporated into the farm's direct expenses. Conversely, if actual wages exceeded the theoretical amount, the difference was recorded as a return to shareholders under other income.

Primary production data were obtained from Lactanet Canada (DHI), which also supplied herd files for DairyComp 305 (V 24.9.1051, Valley Ag software, https://vas.com/). The authors selected 16 potential explanatory production variables based on personal experience, and these included average daily ECM (in kg; 3.5% ECM calculated as [12.82 × kg fat] + [7.13 × kg protein] + [0.323 × kg milk]); average 305-d production (**305M**); average annual SCC; annual linear score (**LS**); average annual DIM; pregnancy rate at a standardized 50-d voluntary wait period (**PR**); biological culls within 60 d in milk (sold due to poor production or died); week 4 milk production (**W4MK**); percentage of herd dry during 45 to 75 d postpartum (**% Dry 45–75**); herd turnover rate (%); lifetime daily yield (**LTY**); herd age; Lifetime Performance Index (LPI); Pro$; age at first calving; and heifer-to-cow ratio.

Milk recording data were used to determine the annual farm production, with adjustments made for milk fed to calves as documented in an enrolment survey. Milk sold was estimated based on revenue, and the ratio of milk produced to milk sold was computed.

Participating farms were asked to supply 5 years of annual financial statements, but only 262 records (farms times years) were available. Each year was treated as a data point, or farm-year. From the original 262 records, 21 farm-years were removed due to missing production data (no DHI), and 71 were excluded because they earned less than 90% of their income from dairy (milk, salvage, calves, and replacement sales). Eight farm-years with less than 95% Holsteins that year were also eliminated to reduce breed-related confounding effects. Additionally, 13 farm-years were excluded where the milk produced differed from the amount sold by more than 120%. In total, 108 farm-years were removed, leaving 42 herds and 149 herd-years for analysis.

Collinearity was tested among the production and operational independent variables (herd size, labor, and purchased feed/kg of butterfat quota) using STATA 17.0 (StataCorp LLC, College Station, TX). The independent variables ECM and 305M (r = 0.94) were highly collinear (r > 0.80), as were ECM and W4MK (r = 0.85), and ECM and LTY (r = 0.81). Week 4 milk production and LTY were removed from the analysis because other transition-related and age metrics were not collinear with ECM. Average 305-d production was considered redundant with ECM and was removed from the analysis. Average annual SCC and LS showed high collinearity (r = 0.90); therefore, LS was retained for further study. Surprisingly, herd age and turnover rate were not highly correlated (r = 0.49). The mean study data for both were similar to those reported elsewhere for the same period (CDIC, 2021). We are unable to explain this lack of collinearity. Normality was assessed with the Shapiro–Wilk test. Data were transformed for herd size, and quadratic terms were included for Pro$ and ECM. The square root was used for purchased feed/kg of butterfat quota. Data from the other variables were winsorized at the first and 99th percentiles to reduce the influence of outliers. Instead of grouping herds by size, the total number of adult cattle was used as a variable to preserve degrees of freedom. The investigation of multiple independent variables in a regression may increase the risk of false-positive results; thus, a Benjamini–Hochberg (**BH**) false discovery rate (**FDR**) procedure was employed (Benjamini and Hochberg, 1995). The association between the remaining 17 variables (including 2 quadratic terms) and EBITDA/kg were screened using univariate linear regression. Applying the BH FDR procedure at α = 0.1 across 17 tests, the largest *P*-value meeting the BH criterion was 0.047; thus, results with *P* ≤ 0.05 were deemed significant for screening. Variables were clustered by farm and year using CLUSTER2 to control for possible repeated-measures issues (Petersen, 2009).

Backward stepwise multivariable linear regression was employed to analyze the association between EBITDA/kg and the remaining independent variables. Under the BH FDR adjustment (α = 0.05; 17 tests), the effective significance threshold was reduced from 0.05 to ∼0.02 in the final model.

Table 1 reports the results of the final multivariable regression. Associations were found between EBITDA/kg and DIM (*P* < 0.001) and between EBITDA/kg and % Dry 45–75 (*P* < 0.001). Labor as a percentage of revenue (*P* < 0.001) and purchased feed/kg quota (*P* < 0.001) were also associated with EBITDA/kg.

**Table 1:** Multivariable linear regression of factors associated with EBITDA/kg, based on data from 42 Canadian herds for the years 2017–2021; this final model accounts for 55% of the variance in EBITDA/kg, with N (herd-years) = 157, adjusted R^2^ = 0.55, and *F* (4, 156) = 45, *P* < 0.001

Variable	Regression coefficient	SE	*P*-value^1^
Intercept	8,167.74	818.15	<0.001
Wage %	−89.07	19.04	<0.001
DIM	−14.81	2.08	<0.001
% Days dry 45–75	7.80	2.39	<0.001
Purchased feed/kg (square root)	−72.03	14.34	<0.001

1 Using the Benjamini–Hochberg false discovery rate procedure at α = 0.05 across 17 tests, the largest *P*-value meeting the BH criterion was 0.0198; thus, results with *P* ≤ 0.02 were deemed significant. Variables that were not significant were not reported to improve table conciseness.

Surprisingly, milk production per cow on an energy-corrected basis was not associated with EBITDA/kg of quota (ECM *P* = 0.12; ECM^2^ *P* = 0.12; r = −0.06; R^2^ = 0.02). Graphically, there is a trend for farms with extreme ECM levels to have higher EBITDA/kg, but this was not significant (Figure 1).

**Figure 1:**
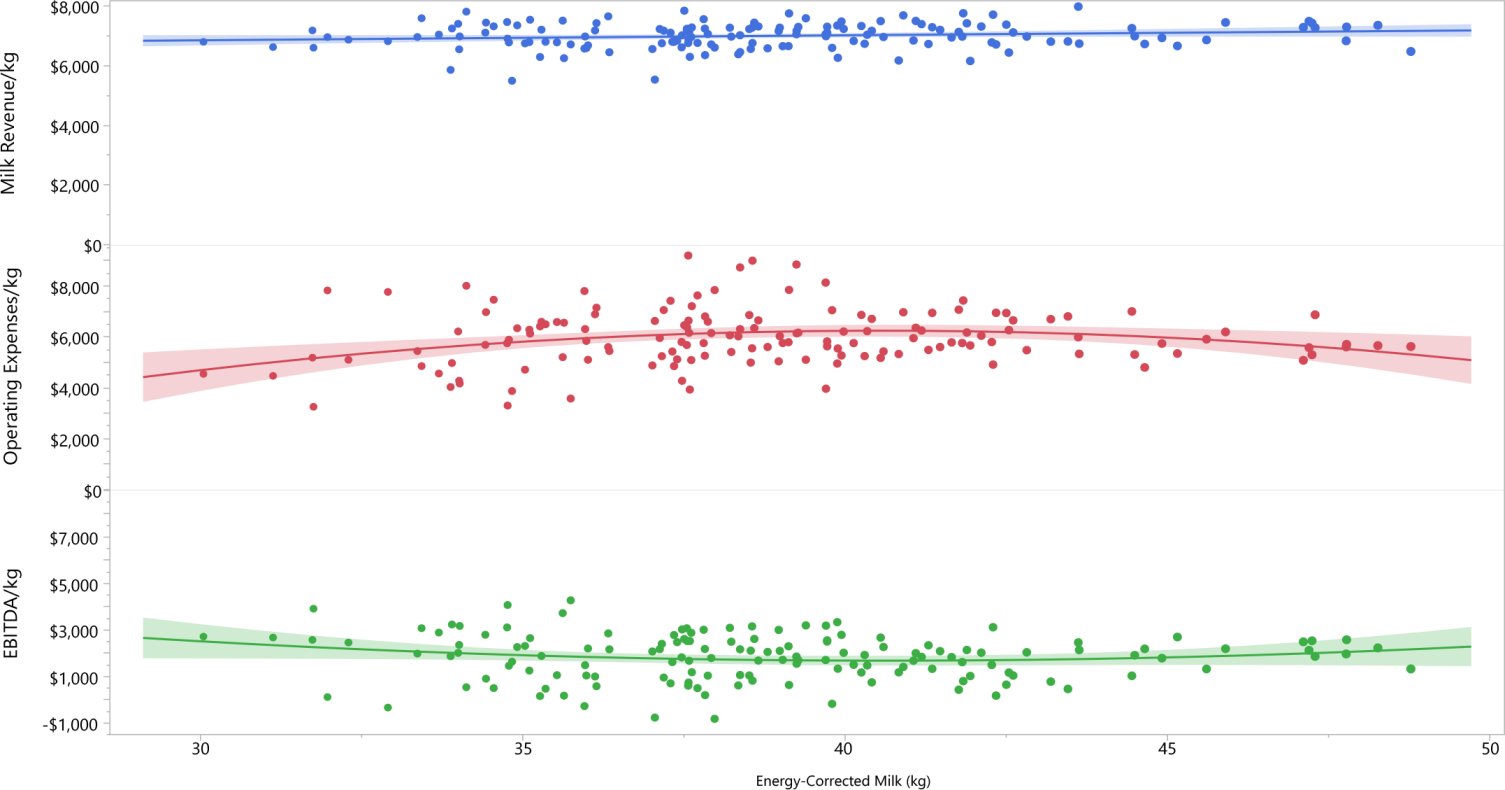
Revenue, operating expenses, and earnings before interest, taxes, depreciation, and amortization (EBITDA) per kilogram of quota ($) compared with the average annual ECM production per cow (kg), based on data from 42 Canadian herds from 2017 to 2021. Each data point represents one herd-year. As expected with supply management, revenue/kg and ECM/cow were not significantly correlated (*P* = 0.08). Operating expenses/kg and ECM/cow were correlated, but the R^2^ value was low (*P* = 0.022, R^2^ = 0.05). EBITDA/kg and ECM/cow were not correlated (*P* = 0.11). Graph prepared using JMP V 17.2 (JMP Statistical Discovery, Cary, NC). The shaded area shows the 95% confidence interval for the fit of the regression line.

Production limiting complicates how advisors assess herd outcomes. From 1972 to 2023, the number of cows required to meet a 100-kg quota (1 kg of fat per cow) in Canada declined from 100 to 72. This transition has gradually decoupled the relevance of the individual cow as the denominator in herd-level comparisons. Quota availability is the most restrictive factor for production expansion, and dividing returns by quota holdings (in kilograms of butterfat) has been broadly adopted by financial advisors. Nevertheless, Canadian production advisors continue to use the individual cow as a denominator; although advantageous in other dairy markets, this practice introduces confusion within the PL context. Farm economic analyses frequently use net farm income (revenues from farming minus direct costs, overhead costs, interest, and depreciation) for financial assessment. Conversely, Canadian financial advisors favor EBITDA, computed by deducting direct and overhead costs from revenue but excluding interest and depreciation, thereby facilitating comparisons among farms with different levels of debt and capital. Accountants may also accelerate depreciation for tax reasons, which could reduce the utility of net farm income as a comparative metric.

Few advisors dispute the use of revenue and expense data to evaluate profitability. Regrettably, most farms conduct this analysis only once annually and generally limit access to financial advisors. Using milk production per cow as an indicator of profit in non-PL markets is founded on the principle of marginality (Vandehaar, 1998). Cows have a maintenance energy requirement independent of production. Once met, any additional energy is directed toward milk production, reproduction, and weight gain. According to the marginal feed-to-milk equation, 1 kg of a typical 2025 lactating dairy ration contains energy for 2.2 ECM of milk (NASEM, 2021). Because marginal milk revenue typically exceeds marginal feed costs, increased production would generally lead to higher profits by diluting maintenance costs. Some have proposed that elevated production levels may necessitate denser, more costly rations due to limitations in DMI or increased passage rates, leading to absorption losses (Bach et al., 2020). Nevertheless, most herds include individual cows that sustain high production on standard rations. Physiological factors, including age and stage of lactation, genetics, and barn management practices, may influence the cow's capacity to convert marginal feed energy into additional milk. The marginal equation of 1:2.2 presumes these elements are standardized. If these are suboptimal, the marginal feed-to-milk ratio may decrease, thereby reducing marginal returns at the individual cow level. Investigating the factors behind these outlier cows under PL conditions provides an opportunity to assess their impact on marginal returns at the herd level.

Average herd DIM was negatively associated with EBITDA/kg (*P* < 0.001). Cows exhibit a declining production curve throughout their lactation (Leon-Velarde et al., 1995). Over time, the maintenance feed requirement remains constant and the efficiency of feed energy conversion to milk diminishes (Vandehaar, 1998; Daniel et al., 2017). Consequently, cows (and herds) with lower DIM demonstrate greater efficiency in converting feed energy into milk production. For instance, consider 2 full sisters housed in the same barn and receiving identical feed—one at 100 DIM, and the other at 200 DIM. The latter will produce less milk. The producer could choose to feed the higher-DIM cow a more concentrated ration; however, if its milk yield is pushed to match that of the first cow, the marginal cost would be higher and production would be more costly due to less efficient energy conversion (Ribeiro et al., 2012). Although this example may appear trivial, it reflects the expectations of many herds with higher DIM toward their feed advisors.

Herd average DIM is generally associated with sound herd reproduction (in year-round calving herds), but cows must also undergo a successful calving transition into lactation to capture future herd production. Short (<40 d) and extended (>70 d) dry periods increase the risk of metabolic disease and culling and decrease future reproduction and production (Olagaray et al., 2020; Overton and Eicker, 2025). We compared the percentage of cows with dry period days between 45 and 75, as these data were readily available, and found a positive association with EBITDA/kg (*P* < 0.001). Although reproduction, culling, and other transition metrics were not separately associated with EBITDA/kg, each contributes to DIM and warrants consideration by advisors.

Our final model identified a negative relationship between EBITDA/kg and purchased feed/kg (*P* < 0.001); however, even after accounting for feed costs, no association was observed between EBITDA and ECM. This is the opposite of the findings of Buza et al. (2014) in a non-PL market, who noted higher feed costs in herds with higher production, but also higher margins. In the PL system, producers and advisors may be tempted to increase milk production at any cost. We recommend that advisors actively participate in calculating feed-to-milk margins to confirm positive returns, rather than simply increasing milk production or lowering costs.

Labor efficiency was evaluated by calculating the total economic wages based on hours worked, divided by total revenue, and expressed as a percentage. The average was 16% (range 5%–39%; SD 6%). The economic wage percentage exhibited a negative correlation with EBITDA/kg (*P* < 0.001), suggesting that financial efficiency declined on farms requiring more hours per unit of milk. Because herd size was also incorporated into the model, this appears to be independent of economies of scale, at least within the herd size range examined in this study (40–700 lactating cows).

This study was limited by (1) the use of income statements prepared for tax purposes, (2) unknown volumes of discarded milk, (3) a limited number of observations, and (4) herds operating in a PL market.

Dairy producers depend on the expertise of production and financial advisors to enhance the productivity and profitability of their herds. Collaboration among advisory groups is crucial; however, this necessitates the adoption of a standardized set of leading (production) and lagging (financial) metrics. Our research underscores the importance of distinguishing between per-cow and per-herd denominators. It is recommended that EBITDA/kg be adopted as the standard financial metric for Canadian advisors. Once financial metrics are established, advisors across all markets should consider the herd's technical efficiency when prioritizing opportunities. Factors affecting efficiency include biological and economic considerations such as DIM, days dry, purchased feed ratios, and labor inputs.
